# Can Deaf Mutes Be Made to Hear and Talk?

**Published:** 1910-12

**Authors:** Maury M. Stapler

**Affiliations:** Macon, Ga.


### CAN DEAF MUTES BE MADE TO HEAR AND TALK?

By Maury Stapler, M. D., Macon, Ga.

A definite affirmative verbal answer to the above question is not all the writer now has to offer the medical profession. He has not only established hearing and speech for more than ten per cent. of the deaf children who have remained in the Macon school for Deafmutes during the past several months, but he is prepared to point out a cause of deafmutism not cited by authorities and to demonstrate that removal of the same is followed by hearing. There are various causes of deafmutism, but it is generally agreed that labyrinthitis is the most frequent one, probably 75 to 80 per cent. The cause of the labyrinthitis has ever been in doubt in about the above percentage of cases. The tests I

made go to prove that adenoids are the prime inciting cause bringing about the labyrinthitis, and consequent deafness, probably after the following manner:

The Eustachian tubes are closed, the air in the middle ear cavities is then pent up and becomes rarified, the pressure of the air on the tympanic membranes gets to be greatest from without, the membranes are pressed into the middle ear, and the ossicular chains forced inward upon the labyrinth.

The stapedius muscles are put upon tension to keep the stapes from being pressed too hard upon the labyrinth, these muscles fail, the persistent pressure having caused loss of their contractil power, the stapes then become dislocated inward and the irritating continuous pressure sets up a labyrinthitis, and this is the labyrinthitis which probably causes children to become deafmutes.

Take a child soon after these conditions are present, remove the adenoids, pull the ossicular chains outward by a suction apparatus just as the stapedii would do if they had the power, and the deafmute child should and does hear in many cases.

After twelve years of theoretical work along this line of though, I established the Macon School for Deafmutes in order to demonstrate to my entire satisfaction and that of others, that the theory would work in practice.

The school is now in its second year. Last year seven deafmutes applied and of that number five were given hearing for words spoken in a slightly raised voice, three of the five kept up treatment for eight months and were taught by an oral teacher. The three acquired a speaking vocabulary of about two hundred words, with which they volunteered to make their wants known. They lost that timidity characteristic of deafmute children, built up their own individualities and are now at their homes, hearing and talking, but guided still by an oral teacher, in order to broaden their vocabularies.

The school opened again on October the 5th, 1910, with seven children taken at random. Except one, all were under five years of age, the one being nine, all girls. After one month in the school, three, who gave a history of congenital deafness, and had no hearing or speech, hear and repeat some words spoken in a loud tone near them. The oldest one, who is nine, was treated by me when she was four and her hearing improved. I had not seen her for four years until she returned to enter school this fall with hearing for words spoken in a very loud tone, and ability to speak three or four words with uncertain distinctness. After a month she shows improvement in both hearing and speech, and is beginning to connect words into sentences and volunteer their use.

The cases are well authenticated, the work having the endorsement of our local medical organization, the Bibb County Medical Society, and the 6th District Medical Association.

One of the children of last year's class and two now in the school, and among those made to hear, are children of physicians of Georgia.

Should it be asked why all those children are not deafmutes whose diseased adenoids close the Eustachian tubes, I should answer that the reason for this seems to lie in the fact that the tubes have to be just sufficiently closed to keep up continuous pressure on the stapedius muscles to overcome their contractil power.

If sufficient air gets into the middle ears every time the child swallows, the pressure would not be sufficiently continuous to overcome the stapedius muscles, the child would only be partially deaf, whereas entire closure of the tubes would cause the tympanic membranes to rupture before the muscles were prostrated, and the stapes would be withdrawn from the labyrinth by the stapedius muscles before a labyrinthitis set up. Were it possible to adjust the closure of the tubes artificially we could probably produce deafmutism in any young child.

Be it well understood that there is no claim to cure all deafmute conditions by the method here set forth. My experience confirms me in the belief, however, that more than ten per cent. of all children attending deafmute schools would become normal citizens if taken at the age of three and treated until they are ten by the methods carried out at the Macon School, that is, remove the adenoids, use the suction massage instrument once a day, have them in a mild climate, under the care of an oral teacher with special directions as to how to make the centers of hearing and speech automatic and co-ordinate, looking strictly to their diet, having one teacher to every five children, teach them tongue gymnastics, until each chlld can lick sweets from the tips of the nose and chin, and can sweep the posterior nares and vault of th pharynx with the tongue. There is no healing lotion that we can apply to the pharynx comparable to the tongue. The ability to clean the pharynx with the tongue is a lost art, to the losing of which is, perhaps, attributable the prevalent amount of tonsil and pharyngeal disease.

### Effects upon the Speech Centers.

The speech centers become dormant in deafmutes, and, although acute hearing be established, the child will not learn to talk; the centers have to be repeatedly aroused by moving the chain of bones in the ear with sufficient force to give something of a shock to the labyrinth. Constant drilling in a loud tone will accomplish the awakening in some cases, but the suction instrument is much more effective. There are many people who have the idea that should a deafmute suddenly get hearing it would immediately begin to talk. Whereas the child has to be taught not only the meaning of words, but the use of sounds. The child has also to have the co-ordinate automatic action of the vocal apparatus which comes from training in order to speak words, more especially sentences.

Those children for whom hearing has been established exhibited the peculiarity of at first transposing the sentences given them to repeat, for instance, when "Hello," Papa," was given, they would reply "Papa, hello." It would seem from this that the ear, like the eye, has to transpose impressions received.

The local cause of deafmutism seems to resolve itself into a case of adenoids, with the sequence of the dislocated ossicular chaina, with resutlant traumatic labyrinthitis, dormant speech and vocal centers in the brain and lack of that concomitant automatic cor-ordination which is developed in a hearing child and translates the aural impressions into spoken words.

Remove the pressure from the labyrinth and keep it removed and nature will attend to the labyrinthitis, and restore at least some of the function of hearing.

### Suction Apparatus and Technique.

The suction apparatus contains a rubber tubing with five branches, and a rubber bag with valves arranged in hard rubber for suction and inflation, with a hard rubber container for medication. The tips of the longest branches close the ears and are held by the patient or assistant, the other two tips close the nostrils, and are held by the left hand of the operator, the suction end of the bag is attached to the remaining tube and held in the right hand of the operator. Suction is made by compressing and relaxing the grip on the bag. The patient shoud keep the cheeks inflated and hold the breath or swallow rapidly while the suction is being made. The ears are being properly reached only when the allae of the nose are drawn down. To inflate, the end of the bag is reversed or a compressed air apparatus may take its place. The patient shutting off the pharynx as before by crying, ballooning the cheeks or swallowing repeatedly. The suction should draw the allae of the nose well down for at least half dozen times at each sitting. The inflation should be under sufficient pressure to overcome the soft palate, which is indicated by a clicking noise in the throat. The ears and nose should be closed with the tips as nearly air tight as possible during suction and inflation. Finally, the palms of the operator's hands should firmly cover the patient's ears and a strong pull upwards should be made on the head. Treatment should be given once a day for some time, depending upon the inflammatory conditions present.

When a deafmute child comes for treatment the name, place of residence, and individual and family history is gotten, tests of the hearing, etc., are made. The next step is an inspection of the ears, nose, throat and general physical conditions; should tympanic membranes appear red and retracted, operate for adenoids to open the Eustachian tubes. In case we find the tympanic members normal and the tubes open. We operate for adenoids, knowing from experience that it is a case where opening and closing of the tubes is intermittent, under favorable conditions, in such cases, the adenoids subside, the tubes allow the passage of some air and the tympanic membranes swing into place. Under unfavorable conditions the tubes are closed and the membranes pressed in, so, in nearly all cases adenoids are present, and after doing the operation the finger is passed into Rosenmuller's fossa, pressing firmly in the direction of the middle ears, then passing the fingers along the mouth of the tubes stripping them, and down the raphae made by the muscle below. The instrumental part of the operation having been done with a curette of the proper size. Following the operation the child's throat is inspected daily for the double purpose of seeing that healing progresses properly, and to make friends with the little fellow, and get co-operation later. If the throat heals and the tympanic membranes remain red, or if a red glow shows through the membranes, palatitive treatment should be instituted, tending to the removal of the inflammation which remains in the middle ear. The treatment followed is gentle suction with my suction apparatus described, followed by inflation with warm menthol vapor and irrigation of the external ear canals with a warm solution of boracic acid. At night a few drops of warm adrenalin solution, of the strength of 1 to 1,000, is dropped into the ears and held in each ear for ten minues, the child being on its side. Should the inflammation not subside in a week, the prognosis for improved hearing is bad. When the inflammation clears away suction is made to reduce the ossicular dislocation. The amount of suction which may be made upon the middle ears is controlled by the soft palate, after a certain amount, air enters from below by pressing the palate up. The rubber suction bag has power to raise the palate in most children. In some cases a pump with more power is used, since no harm can result, the soft palate acting as a safety valve to protect the structure of the ears.

